# Phosphoglycerate mutase 5 exacerbates liver ischemia–reperfusion injury by activating mitochondrial fission

**DOI:** 10.1038/s41598-024-58748-7

**Published:** 2024-04-12

**Authors:** Hongwei Tang, Qiwen Yu, Xu Chen, Jiakai Zhang, Danfeng Guo, Wenzhi Guo, Shuijun Zhang, Xiaoyi Shi

**Affiliations:** 1 Department of Hepatobiliary and Pancreatic Surgery, The First Affiliated Hospital of Zhengzhou University, No.1, East Jianshe Road, Zhengzhou, 450052 Henan China; 2 Henan Engineering Technology Research Center of Organ Transplantation, Zhengzhou, 450052 Henan China; 3 ZhengZhou Engineering Laboratory of Organ Transplantation Technique and Application, Zhengzhou, 450052 Henan China

**Keywords:** PGAM5, Liver I/R injury, Mitochondrial quality control, Mitochondrial fission, Hepatology, Cell death

## Abstract

Although the death of hepatocytes is a crucial trigger of liver ischemia–reperfusion (I/R) injury, the regulation of liver I/R-induced hepatocyte death is still poorly understood. Phosphoglycerate mutase 5 (PGAM5), a mitochondrial Serine/Threonine protein phosphatase, regulates mitochondrial dynamics and is involved in the process of both apoptosis and necrotic. However, it is still unclear what role PGAM5 plays in the death of hepatocytes induced by I/R. Using a PGAM5-silence mice model, we investigated the role of PGAM5 in liver I/R injury and its relevant molecular mechanisms. Our data showed that PGAM5 was highly expressed in mice with liver I/R injury. Silence of PGAM5 could decrease I/R-induced hepatocyte death in mice. In subcellular levels, the silence of PGAM5 could restore mitochondrial membrane potential, increase mitochondrial DNA copy number and transcription levels, inhibit ROS generation, and prevent I/R-induced opening of abnormal mPTP. As for the molecular mechanisms, we indicated that the silence of PGAM5 could inhibit Drp1(S616) phosphorylation, leading to a partial reduction of mitochondrial fission. In addition, Mdivi-1 could inhibit mitochondrial fission, decrease hepatocyte death, and attenuate liver I/R injury in mice. In conclusion, our data reveal the molecular mechanism of PGAM5 in driving hepatocyte death through activating mitochondrial fission in liver I/R injury.

## Introduction

Ischemia–reperfusion (I/R) is an inevitable procedure of liver surgeries, particularly in liver transplantation, and also an important factor affecting the prognosis of patients^1^. I/R-related tissue damage accounts for about 10% of early allograft failure and contributes to the major cause of liver dysfunction after transplantation^2^. Despite its clinical importance, effective therapeutic strategies to protect against liver I/R injury are unavailable mainly due to the complex mechanisms of I/R injury. It is widely believed that the loss of hepatocytes is the basic pathogenic mechanism of liver I/R injury^3^. Apoptosis and necrosis are the two major forms of cell death during I/R injury. With rigorous criteria of identification, Gujral et al.^4^ indicated that apoptosis accounted for only a small fraction of cell death during I/R injury. However, necrosis becomes the main form of cell death, accounting for more than 90% of total cell death^5^. Even though necrosis is a histological feature of liver I/R injury, the precise mechanism regulating necrosis remains unclear.

Mitochondria are highly dynamic double-membrane organelles that undergo constant fission and fusion according to the energy status of the cell^6,7^. When faced with the challenge of bioenergetic or oxidative, mitochondria exhibit functional and structural changes through activating different molecular machinery that regulates mitochondrial fission and fusion. Mitochondrial fission was found to be a critical step of mitochondrial-dependent apoptosis, while mitochondrial fusion is linked to mitochondrial metabolic state^8^. Hypoxia could break up the imbalance between mitochondrial fission and fusion in the rat brain hippocampus^9^. Blocking mitochondrial fission with Mdivi-1 could alleviate cardiac I/R injury in murine^10^. However, it is still unclear how these molecules perceive intracellular pressure and how they coordinate at the molecular level.

Recently, PGAM5 was recognized to give play to the vital effect on mitochondrial homeostasis and cell apoptosis^11–13^. Mitochondrial dysregulation has been confirmed to be implicated in multiple liver diseases^14^. Based on the context, PGAM5 modulates mitochondrial dynamics through two opposite processes: fission and mitophagy^11,12,15^. Mitochondrial fission produces small spherical mitochondria and promotes a main mitochondrial cristae remodeling, and it is characterized by rupturing of the mitochondrial membrane and the disappearance of cristae^16^. Mitochondrial fission in mammalian cells is regulated by a dynamin-related protein 1 (Drp1), and its receptors Fis1 and Mff, while mitochondrial fusion is modulated by Optic atrophy protein 1 (OPA1), mitofusin 1 (MFN1) and mitofusin 2 (MFN2)^17^. Drp-1 is a GTPase, which performs a crucial part in mitochondrial fission. As activated by phosphorylation of Drp1 at Ser616, Drp1 translocations to the discontinuous points at the cleavage sites, where it induces the mitochondrial fission process^18,19^. During the ConA-induced acute liver injury model, PGAM5 expression is upregulated and activates Drp-1 that drives hepatocyte necrosis leading to tissue damage. Correspondingly, PGAM5 was suggested as a crucial regulator of several necrotic pathways because its deficiency could prevent cell necrosis caused by calcium overload and ROS^11^. However, it is still not clear how the fundamental mechanisms of PGAM5 regulate liver I/R injury.

Here, we preliminarily demonstrated that pgam5-drp1-induced mitochondrial fission promotes liver I/R injury to some extent. Using PGAM5 silencing models, we showed that PGAM5-mediated phosphorylation of Drp1 is essential for liver I/R injury. Collectively, we indicated that the PGAM5-Drp1-mitochondrial fission axis plays a crucial role in the context of liver I/R injury.

## Results

### Upregulation of PGAM5 in liver I/R injury

**Figure 1:**
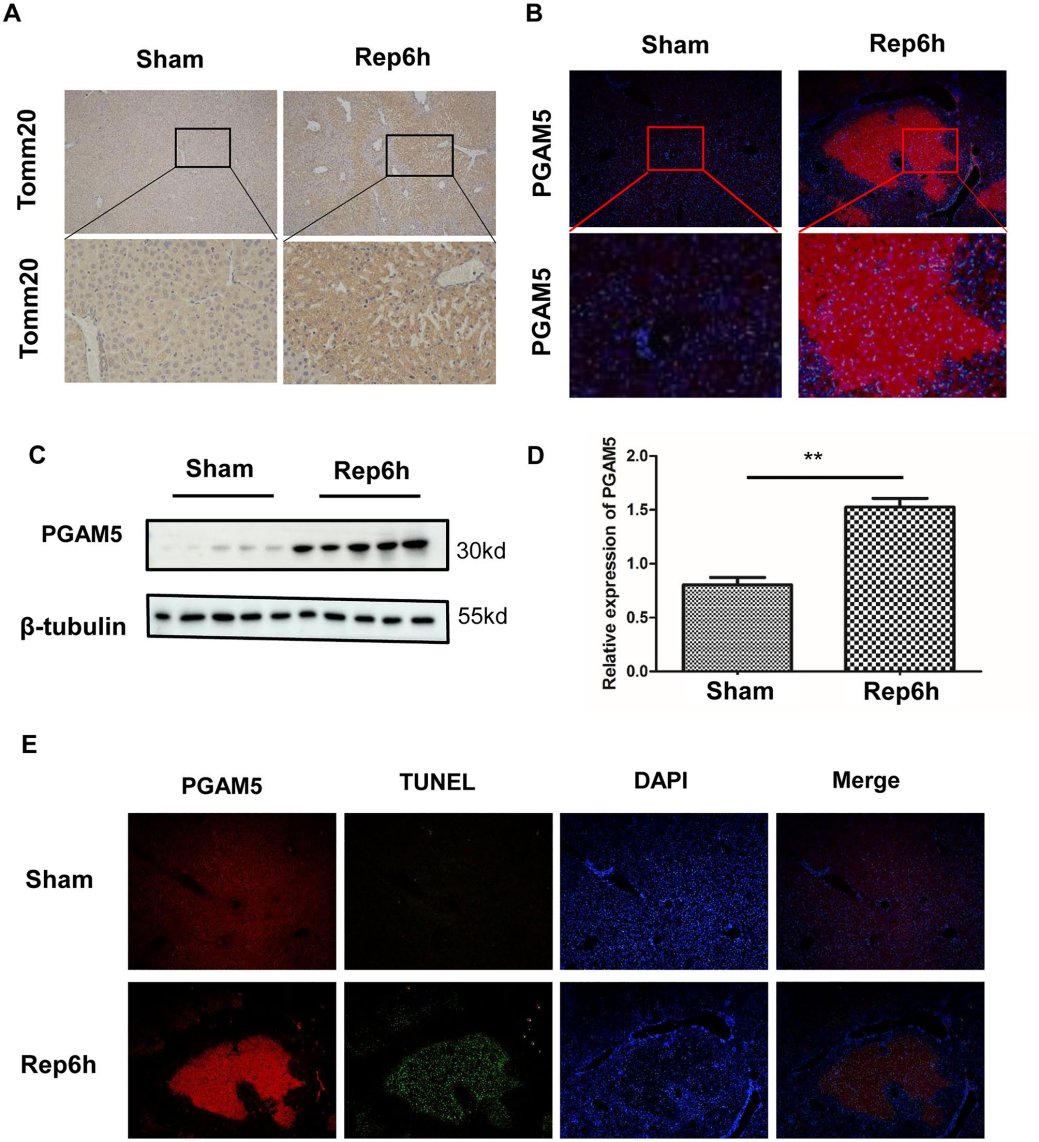
Mitochondrial alterations and elevation of PGAM5 expression in mice liver I/R injury. Data were collected from Sham and Rep 6 h mice, and samples were obtained 6 h of reperfusion for the following analysis. (**A**) Tomm20 immunohistochemical staining of liver transections. Upper panel, 100× magnification. (**B**) Representative immunofluorescence staining pictures of PGAM5 in mice liver sections. Upper panel, 100× magnification. (**C**,**D**) Western blot for PGAM5 protein in mice liver tissues. β-Tubulin functioned as a loading control.And the relative protein expression was calculated (**E**) Containing PGAM5 (red) and TUNEL(green) in liver cross sections. (Magnification:200X) Sham: sham-operated mice, Rep 6 h: mice were subjected to ischemia for 1 h, followed by reperfusion for 6 h. n = 5 mice or 3 independent cell isolations per group. Statistical analysis showed the mean + SD, **p < 0.01.

To investigate whether liver I/R injury is related to disturbances of mitochondrial function, we first stained liver biopsies with Tom20, an indicator of mitochondrial stress. In a liver I/R injury mouse model, tom20 staining presented significant perinuclear aggregation of mitochondria (Fig. 1A). Oppositely, mitochondria were evenly distributed in the cytoplasm of hepatocytes in the sham group (Fig. 1A). These findings indicated that I/R-induced liver injury shows an important characteristic of mitochondrial dysfunction. Studies have shown that PGAM5 is involved in the regulation of mitochondrial homeostasis^20,21^. To further investigate the reason for mitochondrial alterations in the mice exposed to I/R, the expression of PGAM5 in liver tissue was analyzed. In comparison with the control group, PCR results presented a significant elevation of the expression of PGAM5 in mice suffering from I/R injury (Fig. 1B). Similarly, western blotting confirmed an increase in PGAM5 protein in liver tissues (Fig. 1C,D). Importantly, co-staining of PGAM5 with TUNEL showed that PGAM5 might be involved in hepatocyte death (Fig. 1E). Taken together, these data showed that mitochondrial dysfunction and the alteration of PGAM5 expression were observed in the liver cells of the mice with I/R injury.

### Silencing PGAM5 protects mice from liver I/R injury

**Figure 2:**
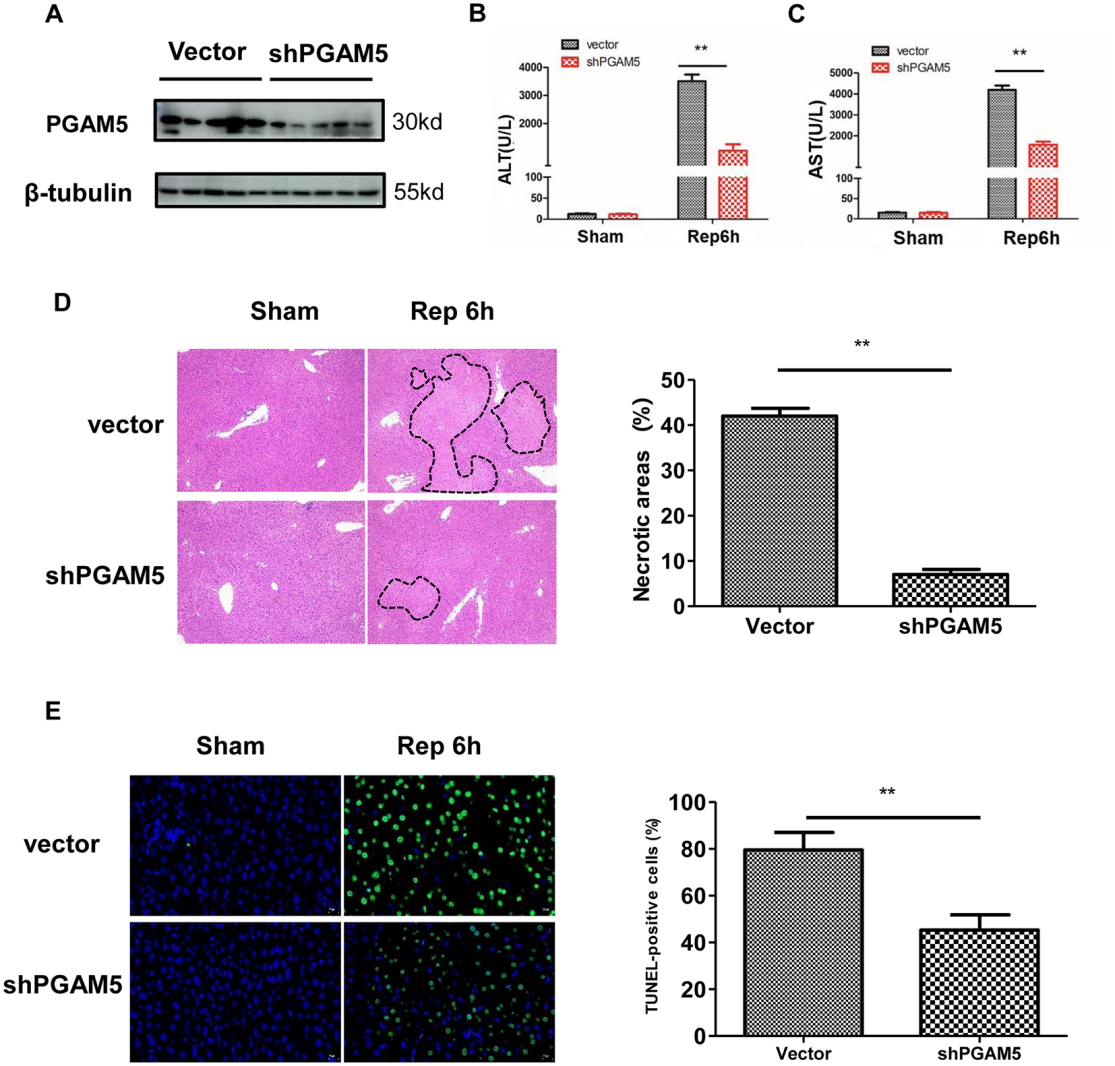
PGAM5 plays an important role in I/R-induced regulatory necrosis of hepatocytes. Data were obtained from Vector and shPGAM5 mice with or without hepatic I/R injury, and samples were collected at 6 h of reperfusion for the following analyses. (**A**) The analysis of PGAM5 Expression in liver tissues by western blotting. β-Tubulin functioned as a loading control. (**B**) Plasma concentrations of ALT. (**C**) Plasma concentrations of AST. (**D**) Representative pictures of H&E staining (Magnification:100X, dashed line shows necrotic area) and statistical analysis of necrotic area (%). (**E**) Representative pictures of TUNEL staining (Magnification:400X, green indicating death cell) and statistical analysis of TUNEL-positive cells (%). Sham: sham-operated mice, Rep 6 h: mice were subjected to ischemia or 1 h, followed by reperfusion for 6 h. n = 5 mice or 3 independent cell isolations per group. Statistical analysis showed the mean + SD, **p < 0.01.

To examine the role of PGAM5 in I/R-induced liver injury, PGAM5-silenced mice were used (Fig. 2A). Correspondingly, both the control mice and PGAM5-silenced mice suffered I/R injury. It was intriguing to note that silencing PGAM5 protected mice from liver I/R injury as demonstrated by the decreased ALT and AST levels (Fig. 2B,C). Additionally, histological analysis of liver sections further confirmed severe tissue injury in vector-treated but not in PGAM5-silenced mice (Fig. 2D). Similarly, a significant reduction of TUNEL-positive cells was observed in PGAM5-silenced mice (Fig. 2E). To investigate the role of PGAM5 in necrosis-driven liver injury, we next treated mice with Necrostatin-1 (Nec-1), a specific necroptosis inhibitor. Anecdotally, the levels of plasma AST and ALT showed that Nec-1 exhibited similar effect of liver damage to that of shPGAM5 (Figure S1A, B). Taken together, these data suggested that PGAM5 is required for hepatocyte necrosis in I/R-induced liver injury.

### PGAM5 deficiency improves AML12 cells mitochondrial function

**Figure 3:**
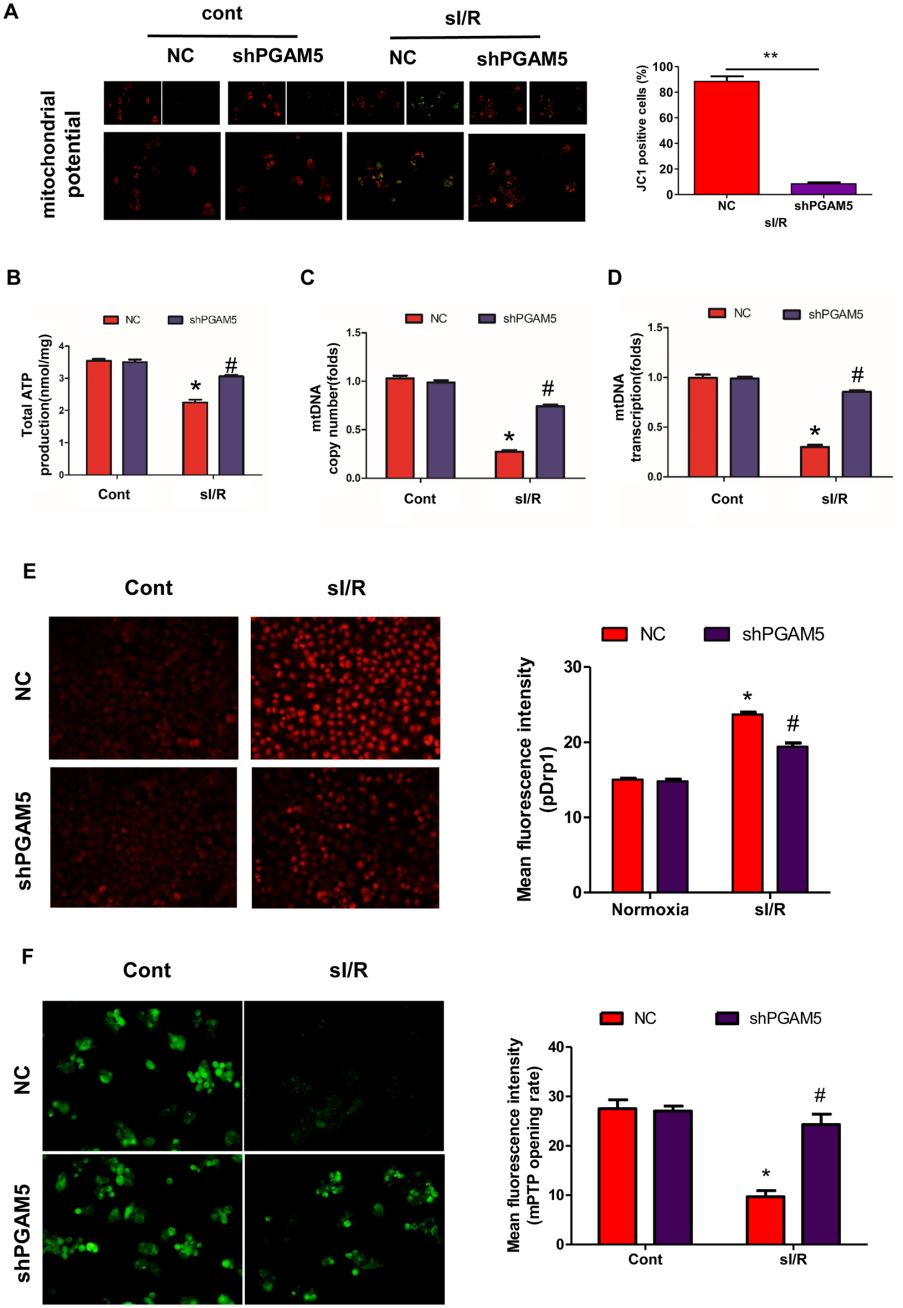
PGAM5 impairs mitochondrial function in AML12 cells. (**A**) Representative images of AML12 cells loaded with the mitochondrial membrane potential indicator JC-1 (Magnification:400X) and statistical analysis of necrotic area (%). (**B**) Measurements of Cellular ATP production in hepatocytes. (**C**) Assessment of mtDNA copy number by complex IV segment. (**D**) The assessment of transcript level of mtDNA by NADH dehydrogenase subunit 1 (ND1). (**E**) Representative images of hepatocytes loaded with the mitochondrial ROS (mtROS) indicator DHE (Magnification:200X) and statistical analysis of mean fluorescence intensity. (**F**) mPTP opening rate was measured through analysis of arbitrary mPTP opening time (Magnification:400X) and statistical analysis of mean fluorescence intensity. Experiments were repeated three times with similar results.. The statistical analysis showed the mean + SD, **p < 0.01. * *p* < 0.05, compared with NC group under Normoxia; # *p* < 0.05, compred with NC group under sI/R.

Mitochondrial quality control has emerged as a key objective for liver protection in I/R-induced liver injury^22,23^. Intriguingly, mitochondrial quality control is finely regulated by PGAM5 via various mechanisms including mitochondrial homeostasis, oxidative stress, and mitophagy^24–26^. These studies encouraged us to study the relationship between PGAM5 and mitochondrial quality control in I/R-induced liver injury. Firstly, by the model of hypoxia/reoxygenation (H/R) injury, we found that H/R treatment significantly increased PGAM5 protein expression in AML12 cells (Figure S2A). Consecutively, we successfully constructed PGAM5 knockdown AML12 cells (Figure S2B). MMP, being an indicator of mitochondrial state, was abolished by sI/R in the NC group but not in the shPGAM5 group (Fig. 3A). Consistently, ATP production was inhibited by sI/R, whereas PGAM5 deficiency was sustained instead (Fig. 3B). Moreover, both mtDNA content (Fig. 3C) and mt-DNA-specific transcript expression (Fig. 3D) were diminished by sI/R. Conversely, sI/R-treated shPGAM5 cells showed partial but significant mtDNA content and transcript level preservation (Fig. 3C,D). In addition, mitochondrial ROS, a by-product of mitochondrial oxidative phosphorylation, was greatly elevated by sI/R in NC cells but kept at a low level after PGAM5 was inhibited (Fig. 3E). Moreover, after sI/R, mPTP opening, a critical event in the transition towards mitochondria-regulated necroptosis, was increased in NC cells but not in shPGAM5 cells (Fig. 3F). Collectively, our data showed that PGAM5 performs an important part in maintaining hepatocyte mitochondrial homeostasis.

### PGAM5 regulates sI/R-induced mitochondrial fission in hepatocytes

**Figure 4:**
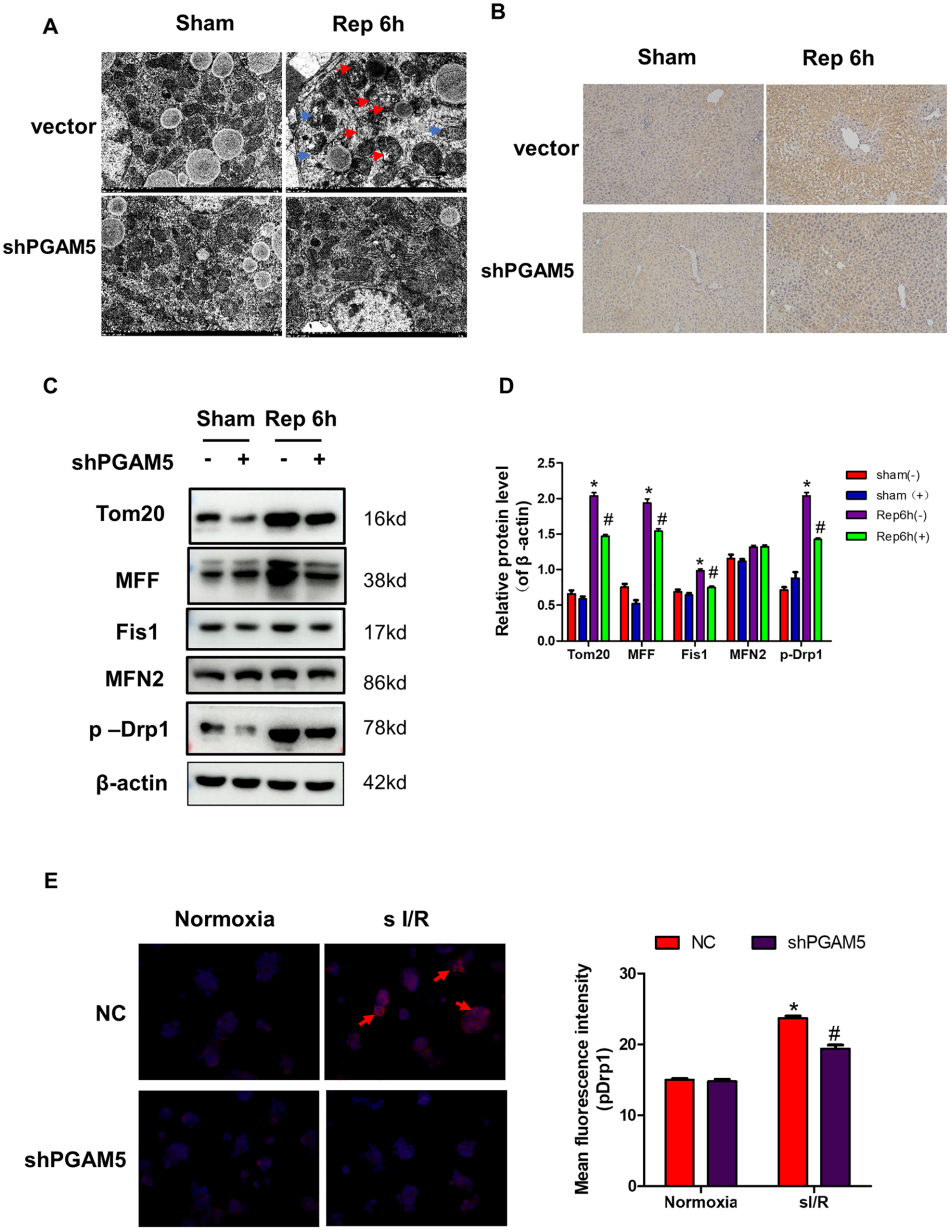
PGAM5 regulates I/R-induced mitochondrial fission in hepatocytes. Data were obtained from Vector and shPGAM5 mice with or without liver I/R injury, and samples were collected at reperfusion for 6 h and used for the following analysis. (**A**) Representative transmission electron microscopic (TEM) pictures (Magnification:10000X) of liver tissues ( red arrows mark mitochondrial with the disappearance of cristae membranes and blue arrows mark mitochondrial membrane rupture). (**B**) Tom20 immunohistochemical staining (Magnification: 200X) in liver cross sections. (**C**) Western blotting of Tom 20, MFF, Fis 1, MFN 2, p-Drp1, and β-actin in liver lysis and (**D**) statistical analysis of protein expression. Western blotting of Tom20 in liver lysis. β-actin worked as a loading control. (**E**) Representative pictures of p-Drp1(Ser 616) staining in AML 12 cells (Magnification: 400X, red arrows mark cells with large pDrp1 foci) and statistical analysis of mean fluorescence intensity. n = 5 mice or 3 independent cell isolations per group. Statistical analysis showed the mean + SD, *p < 0.05, compared with NC group under control group; # *p* < 0.05, compred with group under I/R or sI/R.

We hypothesized that PGAM5 might mediate liver I/R injury by regulating mitochondrial homeostasis. To further test the hypothesis, morphological analysis was performed on mitochondria in liver tissues of I/ R-treated vector mice and shPGAM5 mice. TEM micrographs show that the mitochondrial crest was reduced or absent and the mitochondrial outer membrane was damaged in liver cells from vector mice treated with I/R injury, but these mitochondrial alterations were ameliorated in shPGAM5 mice (Fig. 4A). This observation was confirmed by Tom20 staining ( Fig. 4B). In many instances, excessive mitochondrial fission associated with cell death is accompanied by loss of cristae membranes and mitochondrial fragmentation^27^. We measured the expression of the mitochondrial fission-related proteins pDrp1,MFF, Fis1, and mitochondrial fusion protein MFN2 by western blot. In agreement with our hypothesis, the level of pDrp1,MFF, Fis1, was significantly increased in vector mice subjected to I/R injury, and the inhibition of PGAM5 could reverse these alterations (Fig. 4C,D). Drp1 phosphorylation at the S616 site is a crucial step during the mitochondrial fission process. Firstly, we measured intracellular pDrp1(s616) expression of AML12 cells by immunofluorescent staining and western blot assays. We found that H/R treatment significantly increased intracellular pDrp1(s616) expression , and the inhibition of PGAM5 could block the expression of pDrp1(S616) induced by I/R (Fig. 4D, Figure S3 A ). Taken together, our data showed that Drp1 activation and mitochondrial fission perform a vital part in liver I/R injury and they were regulated by PGAM5.

### restraining of PGAM5-dependent Drp1 activation protects mice from liver I/R injury

**Figure 5:**
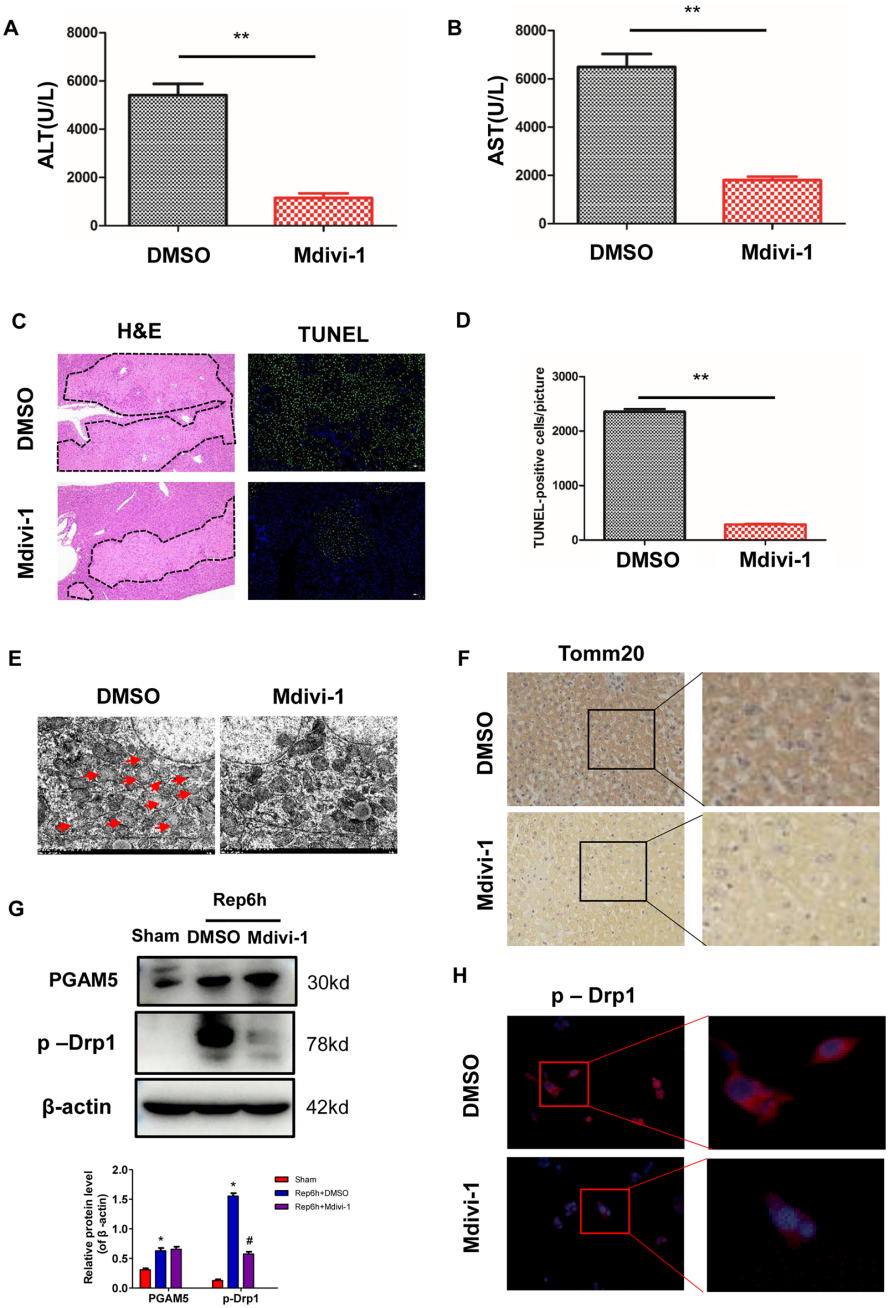
Blocking Drp1 activation keeps mice from liver I/R injury. Pretreatment with Mdivi-1 has a protective effect on I/ R-induced liver injury in mice. Wild-type mice were intraperitoneally injected with 50 mg/kg Mdivi-1 or DMSO as vehicle 30 min before ischemia, and samples were collected for analysis at 6 h of reperfusion. (**A**,**B**) Plasma concentrations of ALT and AST. (**C**) Representative images of H&E staining (Magnification:100X, dashed line shows necrotic area) and TUNEL staining (Magnification:100X, green marking death cell). (**D**) Statistically analyze TUNEL-positive cells. (**E**) Representative TEM pictures of liver tissues (Magnification:10000X, red arrows mark mitochondrial with the disappearance of cristae membranes). (**F**) Tom20 immunohistochemical staining in liver transections. Left panel, 40× magnification. (**G**) Western blotting of PGAM5, pDrp1, and β-actin in liver lysis and statistical analysis of protein expression (**H**) Representative images of p-Drp1(Ser 616) staining AML 12 cells (Magnification: 400X). n = 5 mice or 3 independent cell isolations per group. Statistical analysis indicated the mean + SD, **p < 0.01. *p < 0.05, compared with sham group; # *p* < 0.05, compred with DMSO group under I/R.

Drp1-mediated mitochondrial fission occurs in the early process of apoptosis and necrotic cell death^28^. To further examine whether mitochondrial fission directly contributes to liver injury caused by I/R, Mdivi-1, a specific inhibitor of Drp1, was used. In comparison with DMSO, Mdivi-1 pretreatment significantly reduced plasma ALT and AST levels in WT mice after liver I/R injury (Fig. 5A,B). Additionally, histological analysis and TUNEL staining indicated that pretreated with Mdivi-1 could attenuate hepatic tissue injury and reduce hepatocyte cell death (Fig. 5C,D). Importantly, TEM analysis indicated that Mdivi-1 could suppress mitochondrial fission (Fig. 5E). Immunohistochemical analysis showed that Mdivi-1 could decrease Tom20 aggregates (Fig. 5F) and the formation of large pDrp1(S616) foci (Fig. 5G). Notably, Mdivi-1 did not affect the upregulation of PGAM5, further supporting our previous result that PGAM5 is located upstream of Drp1-mediated mitochondrial fission (Fig. 5F–H, Figure S4A,B). Collectively, our studies suggest that Drp1-mediated mitochondrial fission could drive hepatocyte necrosis and induce hepatic I/R injury.

## Discussion

Liver I/R injury remains a major complication that affects the prognosis of patients, especially in liver transplantation^29^. Despite profound clinical implications, therapies to inhibit I/R at the bedside remain limited due to the complex mechanisms. In addition, the search for an effective strategy to improve liver I/R injury remains an urgent clinical need. Here, we identified PGAM5 as a crucial mediator to mediate I/R hepatic injury in mice. At the molecular level, we further revealed that PGAM5 regulated the activation of Drp1, and then the mitochondrial fission mediated by Drp1 appeared to be a necessary process for necroptosis execution in hepatocytes. Therefore, inhibition of Drp1 significantly ameliorated liver injury induced by I/R.

PGAM5 is a mitochondrial Ser/Thr protein phosphatase that was first identified as a protein interacting with keap1 in eukaryotic cells^30^. According to research, oxidative stress inhibits the ubiquitination of PGAM5 and degradation by ubiquitination, which reveals it comes into play in cellular adaptation to stress^24^. Moreover, despite the lack of data from mammalian cells, interactions between PGAM5 and apoptosis signal-regulated kinase 1(ASK1) have been reported in Drosophila and C. elegans (31). In addition, PGAM5 has been suggested to serve as a key driver of TNFα- mediated necroptosis. This hypothesis is supported by recent studies that PGAM5 conduces to inflammation-associated hepatic cell death^31,32^. Interestingly, studies investigating how PGAM5 functions in liver I/R injury have been rarely reported. Our study indicated that PGAM5 was upregulated in hepatocytes during mice liver I/R injury and this upregulation was restricted to the necrotic area. Similarly, Drp1 phosphorylation induced by I/R and mitochondrial fission were observed in liver cells. Therefore, our data indicated that PGAM5 could regulate mice’s liver I/R injury through a hepatocyte-autonomous mechanism.

Hepatocyte death mediated by I/R is relevant to mitochondrial dysfunction. Studies have shown that ROS, abnormal mitochondrial fission and fusion, and defective mitophagy may induce hepatocyte death through a synergistic multi-effect (34). Our results indicated that liver I/R injury did not induce mitochondrial fission in PGAM5-blocked hepatocytes, an effect associated with PGAM5-mediated Drp1 activation. The phosphorylation of Drp1 at two critical sites leads to both stimulation (Ser616) and inhibition (Ser637) of mitochondrial fission^11^. This inhibition can be reversed by dephosphorylating Ser637, such as with calcineurin^11^. Previous research has shown that PGAM5 can directly induce Ser637 phosphorylation, and activate the GTPase activity (11). After I/R injury, Drp1 is dephosphorylated at Ser 637 due to PGAM5 upregulation, while Ser616 of Drp1 gets phosphorylated in a PGAM5-independent manner, ultimately inducing mitochondrial fission. Notably, several studies have shown that mitochondrial fission is an upstream factor in both apoptosis and necroptosis, although it remains unclear whether necroptosis or necroptosis is induced by PGAM5-independent mitochondrial fission.

As an important pathway of hepatocyte necrosis, the PGAM5-Drp1 axis plays an important role in the pathogenesis of liver I/R injury and has far-reaching therapeutic significance. Mdivi-1, a selective Drp 1 inhibitor, has been used as a promising target to treat the diseases involved in myocardial infarction, stroke, and cerebral I/R injury^33^. Our study indicated that Drp1-mediated mitochondrial fission plays a crucial part in mice’s hepatic I/R injury. Importantly, Mdivi-1 could attenuate liver I/R injury and inhibit liver necrosis in the experimental mice model. Therefore, our research results put forward a kind of interesting possibility, namely Mdivi—1 may be targeted by necrosis of liver cells as a therapeutic agent for liver I/R injury.

In conclusion, our data indicated that PGAM5-mediated necrosis is crucial to I/R-induced liver injury. As a downstream event of PGAM5, Drp1 could initiate hepatocyte necrosis by facilitating mitochondrial fission. This is of clinical significance, as Drp1 inhibitors could be used as effective drugs for liver I/R injury. Alternatively, targeting necrosis might benefit future therapeutic strategies for liver I/R injury.

## Experimental procedures

### Animal procedures

C57BL/6 male mice (23–27 g) were purchased from Weitong Lihua Co. (Beijing, China) and housed in our facility under standard conditions. All animal protocols were approved by the Animal Care and Use Committee of The First Affiliated Hospital of Zhengzhou University (Ethical number: KY-2021-00556, Zhengzhou, China), and the study was conducted in compliance with the ARRIVE guidelines. All methods were performed in accordance with the relevant guidelines and regulations. To inactivate PGAM5, adeno-associated virus (AAV) vectors containing PGAM5-specific shRNA were synthesized by GENECHEM (Shanghai, China) and injected through the mouse tail vein with 1 × 10^12^ V g/mouse. The sequences of shRNA were as follows: CCAACTTCTCAGCTCAATTAA. Three weeks following viral injection, a mice model of hepatic ischemia–reperfusion injury was constructed according to our previous study^34^. Briefly, the liver was exposed via laparotomy, and then, a micro-vessel clip (Fine Science Tools, cat.18055-06) was used to clamp the branches of the portal triad for 60 min followed by 6 h reperfusion. Following the experiment, all mice were euthanized, and serum and liver samples were collected for future experiments.

### Histology, immunohistochemistry, immunofluorescence, and TUNEL

Liver tissues were immobilized with 4% paraformaldehyde and then embedded and sectioned at a thickness of 5 μm. For IHC analysis, the slices were dewaxed and treated with antigen repair, and then were incubated with TOM20 (1:1000, cat. No. 11802-1-AP, Proteintech, Wuhan, China), and followed with a secondary antibody. For Immunofluorescence analysis, the following antibodies were used: PGAM5 (1:200, cat. No. Sc515880, Santa Cruz, CA, USA), p-Drp1(Ser616) (1:200, cat. no. 3455, CST, USA). The nuclei were stained with DAPI. TUNEL staining was conducted by using a FITC Tunel cell apoptosis assay kit (Seville, Wuhan, China) in accordance with the instructions. Besides, it was necessary to capture at least three images from distinct areas for each stained section, and each group had at least three mice. Photoshop CS 5 was used for image analysis.

### Cell culture and sI/R in vitro

Alpha Mouse Liver (AML12) cells were cultured in DMEM/F12 (Gibco, CA, USA) containing 10% fetal bovine serum, 10 μg/ml Insulin, 5.5 μg/ml Transferrin, 5 ng/ml Selenium, and 40 ng/ml Dexamethasone. Cells were then maintained in an incubator with 5% CO_2_ at 37 °C. To satisfy hypoxic conditions, the cells were placed into a tri-gas incubator (Galaxy 48 R, Eppendorf, Hamburg, Germany) with 1% oxygen for 6 h. For reoxygenation, the cells were then transferred to normoxic conditions for the indicated time.

### Western blotting

Proteins were lysed by RIPA lysates (Solarbio, Beijing, China) added with PMSF (Solarbio, Beijing, China) and phosphatase inhibitors (PhosphoStop, Roche, Germany). And then proteins were separated and transferred to PVDF membranes. Membranes were incubated overnight with the following primary antibodies: PGAM5 (1:500, cat.no.sc515880,santa cruz, CA, USA), Tom20(1:8000, cat.no. 11802-1-AP, Proteintech, Wuhan, China), MFF (1:20,000, cat.no. 17090-1-AP, Proteintech, Wuhan, China), Fis1 (1:1000, cat.no. 10956-1-AP, Proteintech, Wuhan, China), MFN2 (1:20,000, cat.no. 12186-1-AP, Proteintech, Wuhan, China), β-actin (1:20,000, cat.no. 20536-1-AP, Proteintech, Wuhan, China), β-tubulin (1:10,000, cat.no. 10094-1-AP, Proteintech, Wuhan, China), and p-Drp1 (1:1000, cat.no. 3455, CST, USA). HRP-labeled secondary antibody was applied at room temperature for 1 h. The chemiluminescence signal was detected using an Amersham Imager 800 (Amersham Biosciences, Buckinghamshire, UK).

### ROS and mitochondrial membrane potential (MMP) measurements

ROS was detected by using Dihydroethidium (DHE, Biyuntian, Shanghai, China) according to the instructions. In short, AML 12 cells were washed 3 times with PBS and then incubated for 30 min at 37 °C with 5 μM DHE. After washing, fluorescence images were observed by an inverted fluorescence microscope (Olympus IX71, Japan). MMP was assessed by JC-1 staining (Biyuntian, Shanghai, China). Briefly, AML 12 cells were washed 3 times with PBS, and incubated for 20 min at 37 °C with DMEM containing 5 μg/ml JC-1 dye. Then, fluorescence images were observed by an inverted fluorescence microscope (Olympus IX71, Japan). Red fluorescence represented higher MMP, while green fluorescence represented lower MMP.

### ATP production and mitochondrial permeability transition pore (mPTP) opening determination

ATP detection kit (Biyuntian, Shanghai, China) was used to detect ATP concentration. In brief, after hypoxia-reoxygenation, the cells were fully lysed by adding 200 μl of cell lysis and then centrifuged for 5 min at 4 °C and 12,000 g to remove insoluble substances. Then the supernatant was collected and incubated with the ATP working solution. Relative light units (RLU) were measured by chemiluminometer (Varioskan lux, Thermol Biotech, USA). The ATP productions were calculated on the basis of the standard curve and expressed as nmol/mg protein.

mPTP opening was tested by an mPTP detection kit in line with the manufacturer’s instructions. In short, after washing with PBS, the AML12 cells were cultured with calcein-AM/CoCl2 working buffer for 30 min at 37 °C. Subsequently, the buffer was removed and replaced with prewarmed fresh medium, and then the cells were further incubated for 30 min at 37 °C. Afterward, the cells were washed 2–3 times with PBS, and the fluorescence was viewed by an inverted fluorescence microscope (Olympus IX71, Japan).

### Gene silencing

The shRNA against PGAM5 was got from Public Protein/Plasmid Library (PPL, Nanjing, China). Cell transfection was performed with Lipo2000 (Invitrogen, Thermo Fisher Scientific, USA) in light of the manufacturer’s instructions. Then the transfected cells were screened by puromycin (2 μg/ml). The transfection efficiency was confirmed by Western blot. The sequences of shRNA were as follows: shRNA1: AACCACTGTCTCTGATCAA, shRNA2: CCTTCCGGAACT.

ACATCCA, shRNA3: CCAAGCTGGACCACTACAAAG.

### Real-time PCR

Total RNA was extracted with TRIzol (CW Bio, Beijing, China). Then the extracted RNA was reverse-transcribed to cDNA applying a NeuScript II 1st strand cDNA synthesis kit (NUOWEIZAN, Nanjing, China). Quantitative real-time PCR was conducted by a 2 × SYBR Green PCR Master Mix (US EVERBRIGHT, Suzhou, China) in line with the manufacturer’s instructions. Relative Fold changes in gene expression were calculated through the ΔCt (cycle threshold) values and normalized to the housekeeping gene (GAPDH). Primers are shown in Table 1.

**Table 1:** Primers for qPCR.

Gene	Forward prime	Reverse prime
PGAM5	5′-ATCTGGAGAAGACGAGTTGACA-3′	5′-CCTGTTCCCGACCTAATGGT -3′
ND-1	5′-ATGGTCAGTCTGTCATGGTGGAAC-3′	5- GCATAGCACAAGCAGCGACAAC-3′
Complex-IV	5′-CAGGATTCTTCTGAGCGTTCTATCA-3′	5′-AATTCCTGTTGGAGGTCAGCA-3′
GAPDH	5′-ACGGCAAATTCAACGGCACAGTCA-3′	5′-TGGGGGCATCGGCAGAAGG-3

### Statistical analysis

Statistical analysis was conducted using SPSS software (version 21.0). The statistical significance was determined by the Student’s t-test. Multiple group comparisons were analyzed using one‐way ANOVA. A *p*-value of < 0.05 was considered statistically significant.

### Supplementary Information

Supplementary Information.

Supplementary Figures.

## Data Availability

The datasets used and/or analyzed during the current study available from the corresponding author on reasonable request.
