# Supplementary material for: Phosphoglycerate mutase 5 exacerbates liver ischemia–reperfusion injury by activating mitochondrial fission
Source: Sci Rep. 2024 Apr 12;14:8535. doi: 10.1038/s41598-024-58748-7 (PMC11014912; doi:10.1038/s41598-024-58748-7)

Panel A represent the western blot data shown in Figure 1

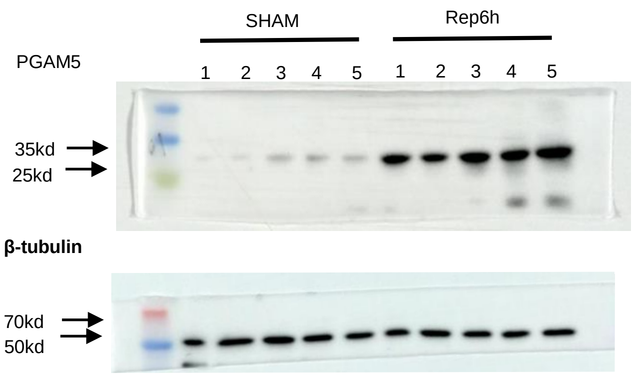

Panel B represent the western blot data shown in Figure 2

Vector shPGAM5

PGAM5

35kd
25kd

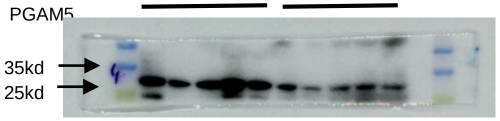

β-tubulin

70kd
50kd

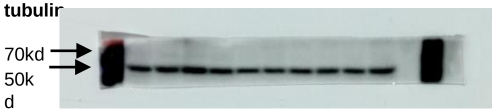

Panel C represent the western blot data shown in Figure 4

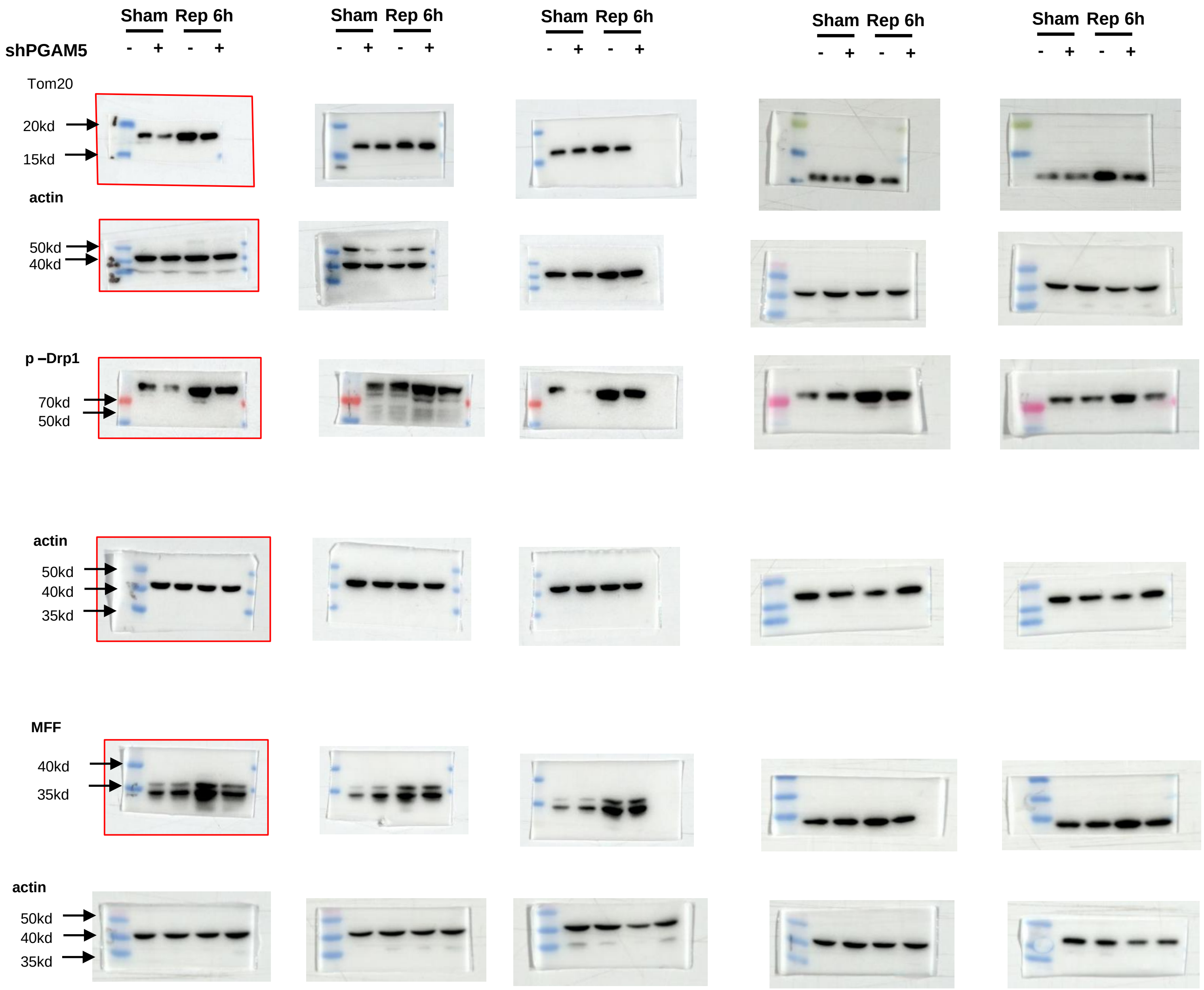

Panel C represent the western blot data shown in Figure 4

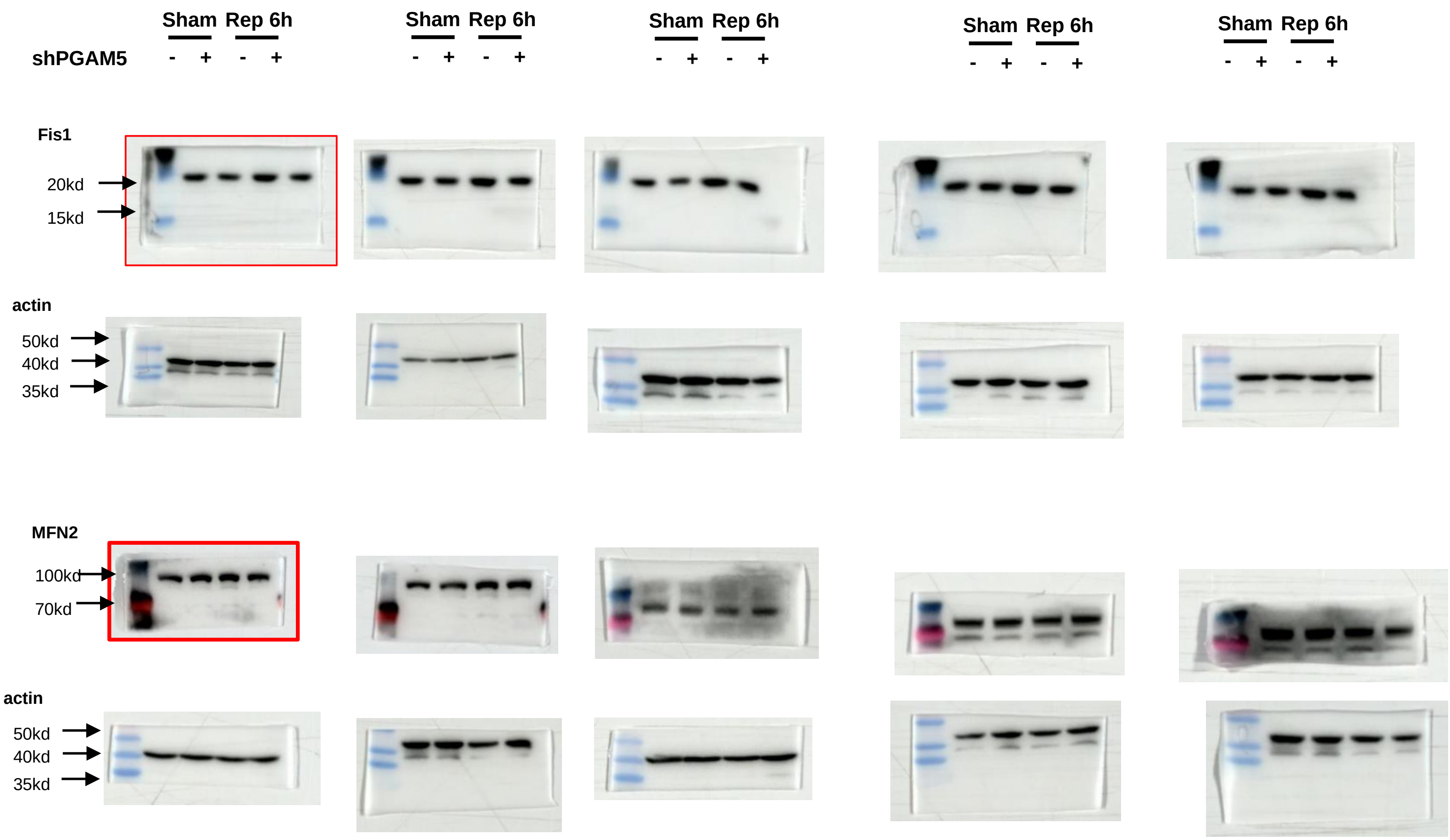

Panel D represent the western
blot data shown in Figure 5

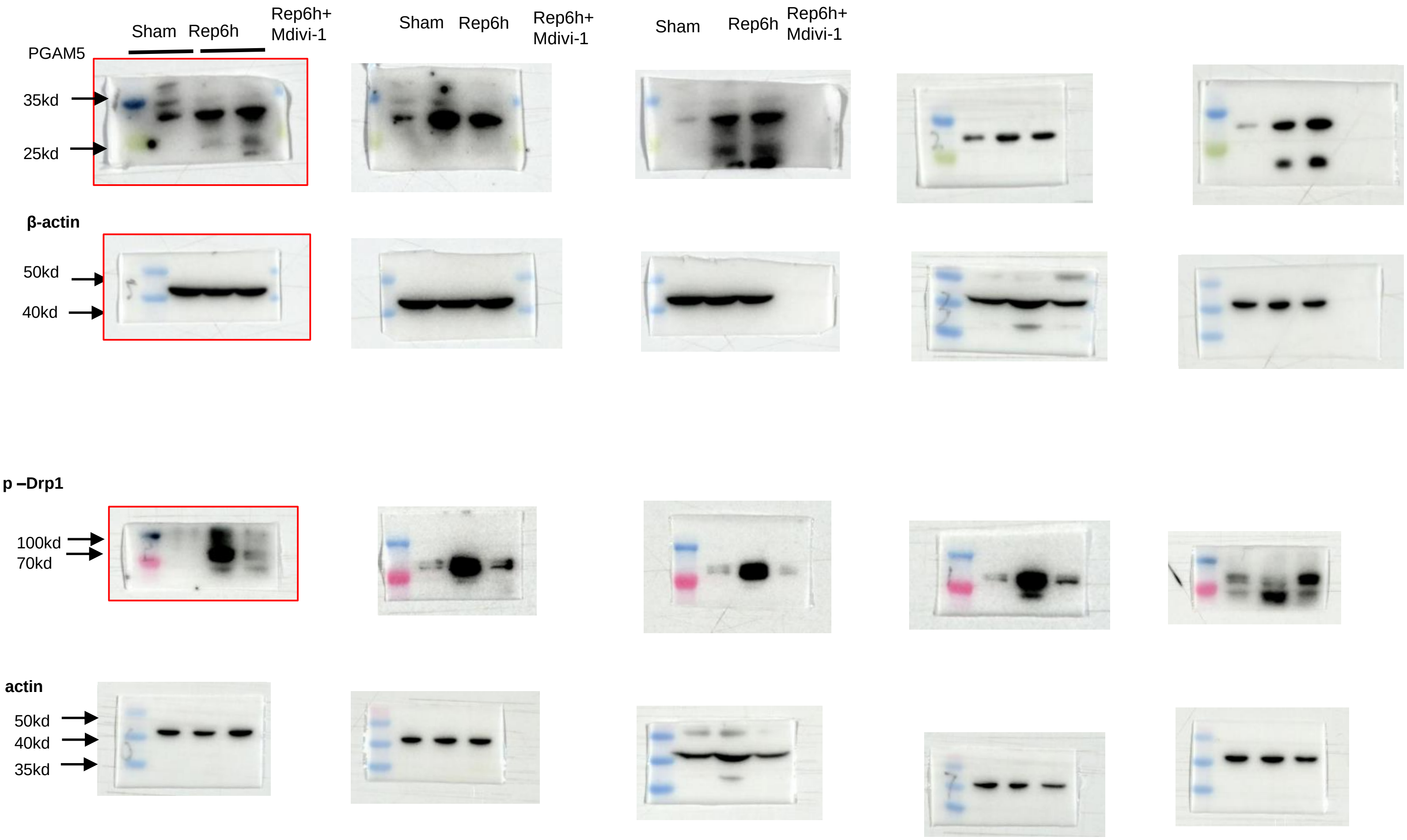

Panel D represent the western blot data shown in Figure s2

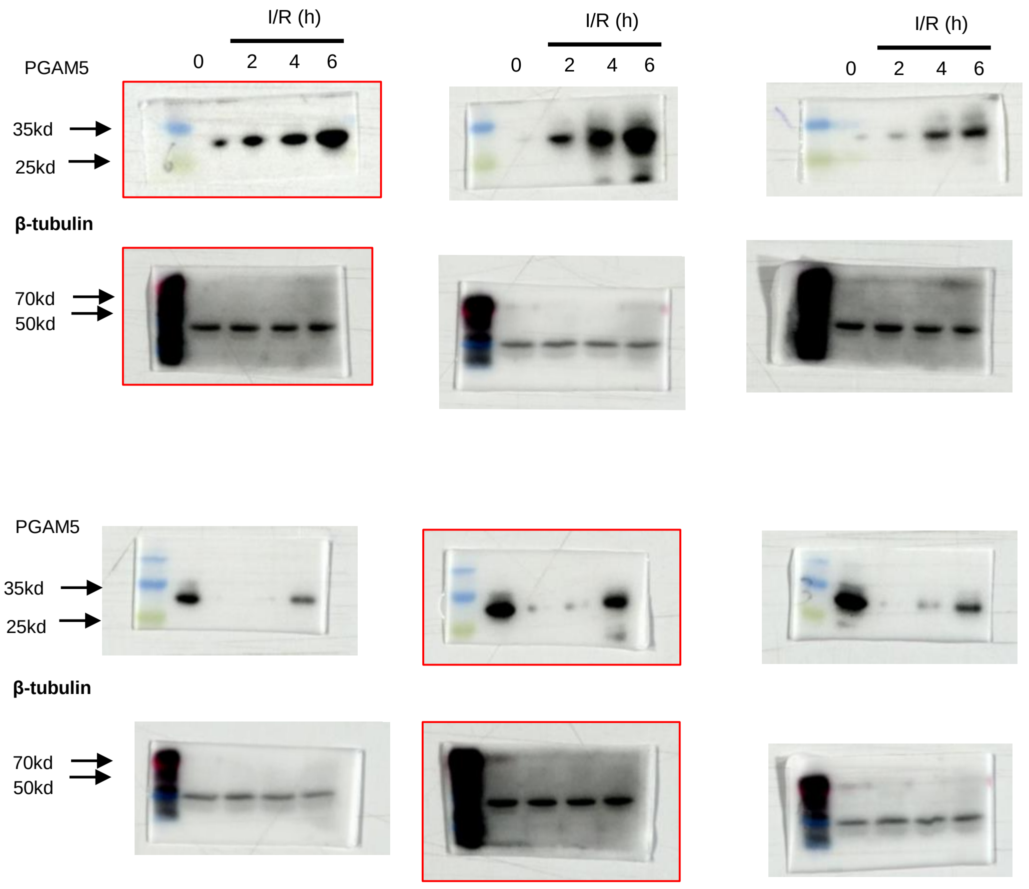

Panel D represent the western blot data shown in Figure s3

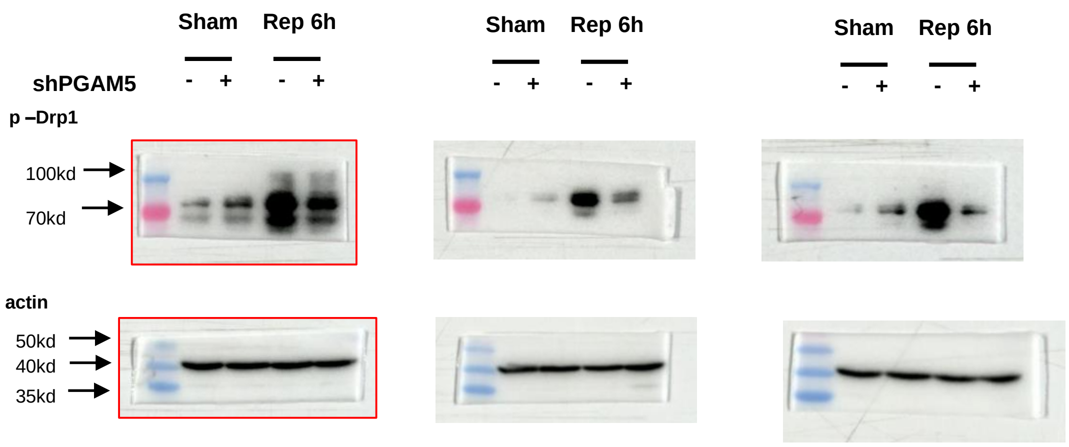

Panel D represent the western blot data shown in Figure s4

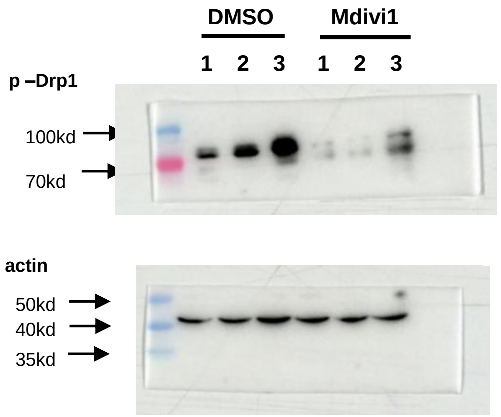

Supplement: Supplementary file 1 — Supplementary Information. [file 41598_2024_58748_MOESM1_ESM.pdf]
