# Supplementary material for: Phosphoglycerate mutase 5 exacerbates liver ischemia–reperfusion injury by activating mitochondrial fission
Source: Sci Rep. 2024 Apr 12;14:8535. doi: 10.1038/s41598-024-58748-7 (PMC11014912; doi:10.1038/s41598-024-58748-7)
Supplement: Supplementary file 2 — Supplementary Figures. [file 41598_2024_58748_MOESM2_ESM.pdf]

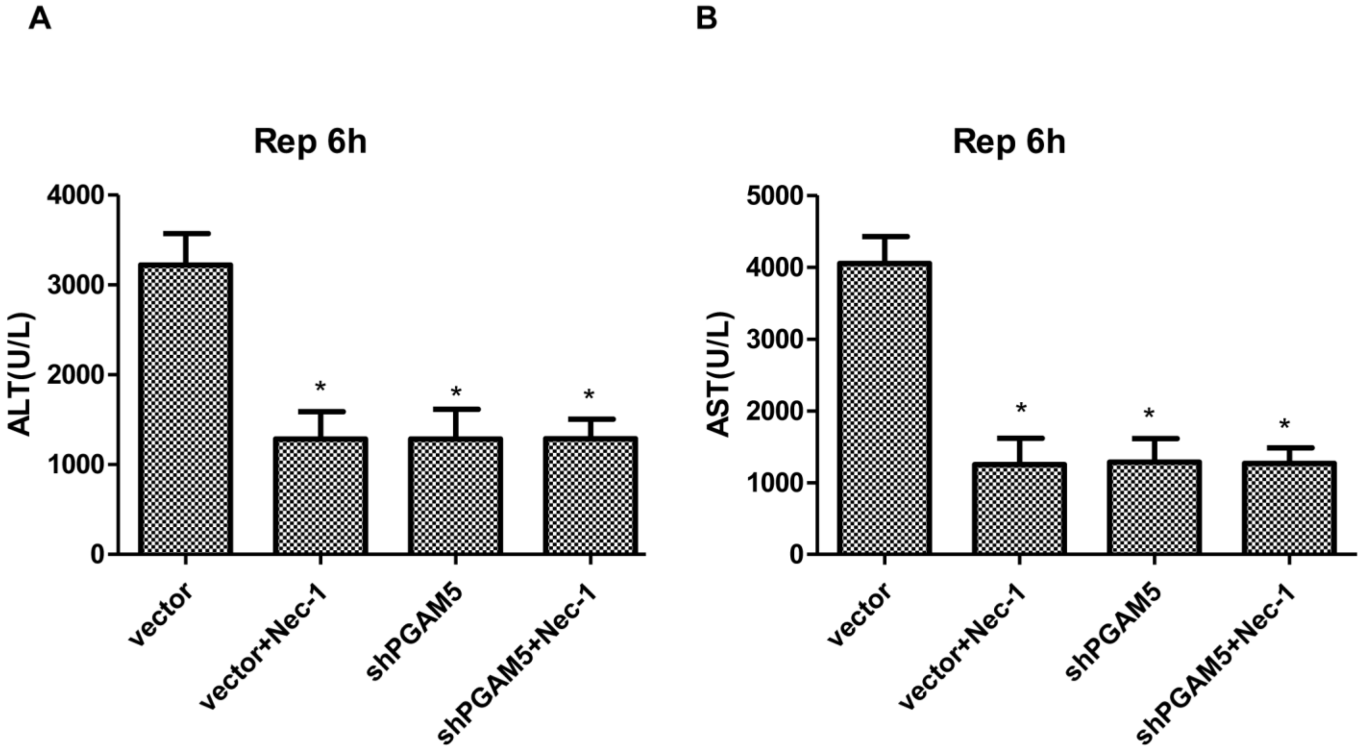

Figure S1 Nec-1 exhibited similar effect of liver damage to that of shPGAM5

(A-B) Plasma concentrations of ALT and AST. * $p<0.05$, compared with vector group

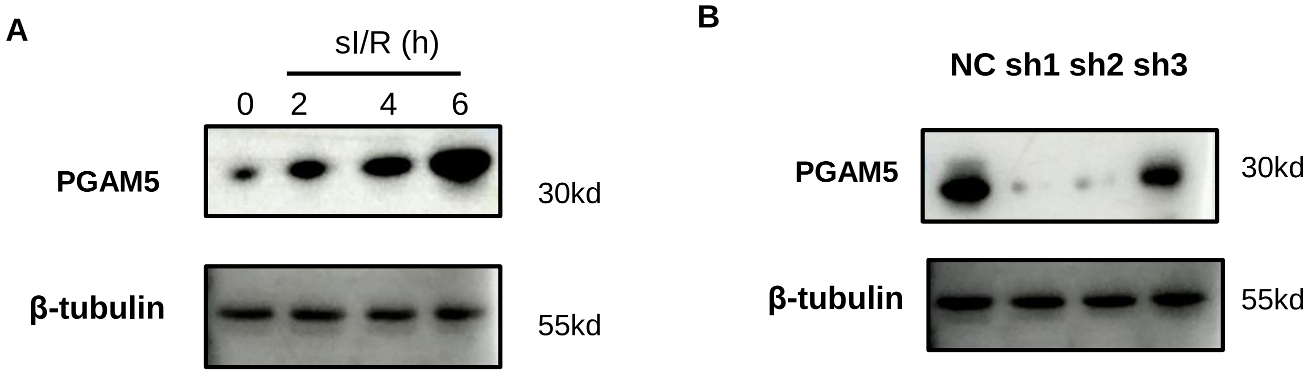

Figure S2 The elevation of PGAM5 expression in AML12 cells subjected to sI/R injury

(A) AML12 cells were exposed to hypoxia injury for 6 h followed by the indicated time of reoxygenation, and the expression of PGAM5 was detected by western blot. (B) The silencing efficiency of PGAM5 was assessed by means of Western Blot analysis.

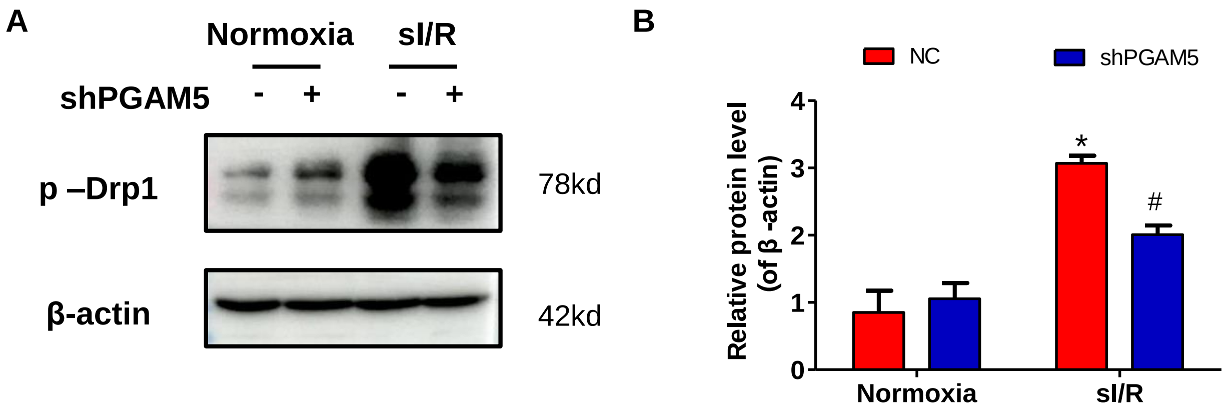

Figure S3 PGAM5 regulates I/R-induced mitochondrial fission in hepatocytes.

(A) Western blotting of p-Drp1 and β-actin in AML12 cells and (B) statistical analysis of protein expression. * $p<0.05$, compared with NC group under Normoxia; # $p<0.05$, compred with NC group under sI/R.

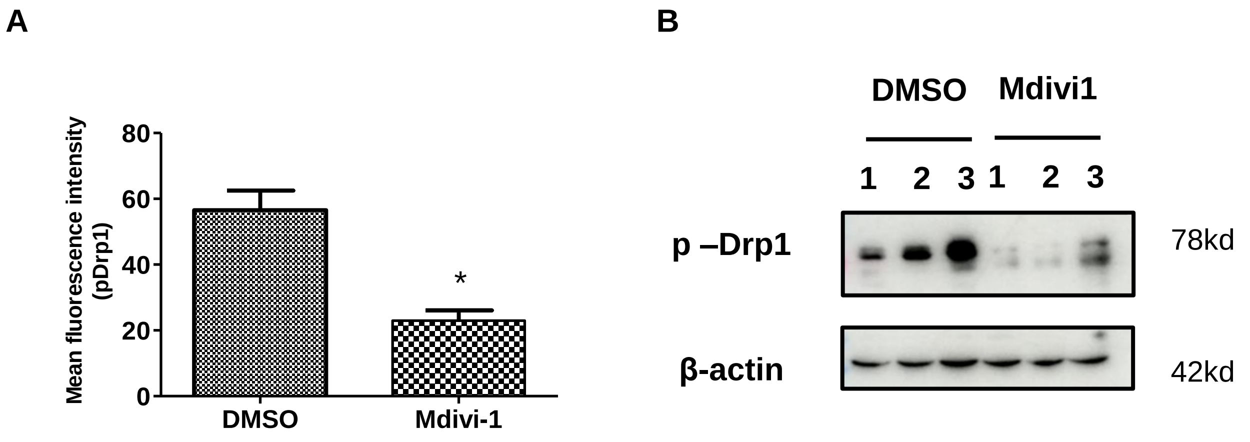

Figure S4 Blocking Drp1 activation keeps mice from H/R injury. (A) Statistical analysis of fluorescence intensity of p-Drp1 in AML12 cells when treated with or without Mdivi1. (B) Western blotting of p-Drp1 and β-actin in AML12 cells when treated with or without Mdivi1. * $p<0.05$, compared with the DMSO group under I/R.
